# Novel Mutation of SARS-CoV-2, Vietnam, July 2020

**DOI:** 10.3201/eid2705.210013

**Published:** 2021-05

**Authors:** Hoang Vu Mai Phuong, Trinh Son Tung, Ung Thi Hong Trang, Nguyen Le Khanh Hang, Nguyen Vu Son, Pham thi Hien, Le thi Thanh, Vuong Duc Cuong, Ton That Thanh, Nguyen thi Thanh Nhan, Tran Nhu Duong, Ngu Duy Nghia, Tran Anh Tu, Marc Choisy, Maia A. Rabaa, H. Rogier van Doorn, Dang Duc Anh, Le Quynh Mai

**Affiliations:** National Institute of Hygiene and Epidemiology, Hanoi, Vietnam (H.V.M. Phuong, U.T.H. Trang, N.L.K. Hang, N.V. Son, P. thi Hien, L. thi Thanh, V.D. Cuong, T.N. Duong, N.D. Nghia, T.A. Tu, D.D. Anh, L.Q. Mai);; Oxford University Clinical Research Unit, Hanoi (T.S. Tung, H.R. van Doorn);; Centre for Disease Control and Prevention, Danang, Vietnam (T.T. Thanh, N. thi T. Nhan);; Oxford University Clinical Research Unit, Ho Chi Minh City, Vietnam (M. Choisy, M.A. Rabaa);; University of Oxford, Oxford, UK (M. Choisy, M.A. Rabaa, H.R. van Doorn)

**Keywords:** COVID-19, 2019 novel coronavirus disease, SARS-CoV-2, severe acute respiratory syndrome coronavirus 2, viruses, respiratory infections, zoonoses, molecular epidemiology, reintroduction, Danang, Vietnam, coronavirus disease

## Abstract

A cluster of severe acute respiratory syndrome coronavirus 2 infections in Danang, Vietnam, began July 25, 2020, and resulted in 551 confirmed cases and 35 deaths as of February 2021. We analyzed 26 sequences from this cluster and identified a novel shared mutation in nonstructural protein 9, suggesting a single introduction into Vietnam.

Vietnam experienced 2 clusters of severe acute respiratory syndrome coronavirus 2 (SARS-CoV-2) infections during January 23–April 15, 2020 (270 cases, 163/270 imported) (*1*–*4*). After 99 days without community transmission, a cluster of SARS-CoV-2 infections of unknown origin was detected in Danang; it was found in Danang General Hospital on July 25 in a 57-year-old male patient (DN001) experiencing pneumonia who had no travel history. During a subsequent round of contact tracing, 14 additional SARS-CoV-2–positive cases were detected both in the community (n = 3) and Danang hospitals (n = 11). Vietnam then initiated large-scale contact tracing and quarantining. A total of 551 confirmed cases were reported from 15 cities and provinces across the country; 540 (98%) either were related to major hospitals in Danang or were in patients who had visited Danang during July 25–September 3. This cluster included 35 COVID-19 fatalities, most (32) hospital-acquired and associated with concurrent conditions or old age (*2*,*5*). Danang General Hospital, the epicenter of the outbreak, reported 246 cases among inpatients, caregivers, and healthcare workers. Strict prevention measures of contact tracing, quarantine, and isolation were again implemented nationally, and the outbreak was successfully contained. We describe the molecular epidemiology of this cluster.

We performed sequencing of 26 nasopharyngeal or throat swab specimens that were sent to the National Institute of Hygiene and Epidemiology (Hanoi, Vietnam) for diagnostics; all were positive for SARS-CoV-2 and had cycle threshold value <30 by real-time reverse transcription PCR (*6**–**8*). Of those specimens, 18 were collected from patients in hospitals and communities in Danang and the rest from outside Danang: Ha Nam (1), Quang Nam (1), Thanh Hoa (1), Hanoi (2), Lang Son (2), and Hai Duong (1). We uploaded sequences to the GISAID database (https://www.gisaid.org; accession nos. 759869–91 and 766029–31).

All 26 sequences belonged to lineage B.1.1 and clustered together in the global tree, with the exception of DN013 (Figure, panel A). DN013 contains an additional single-nucleotide polymorphism (SNP), C835T, that is also present in sequences from India and that artifactually clusters with those sequences in the global tree (Figure, panel A). A novel SNP at position 12772 (A>C) was found in all 26 sequences; it represents a nonsynonymous mutation in nonstructural protein 9 resulting in a leucine to phenylalanine change at amino acid site 4169 in open reading frame 1ab. This SNP was not reported in any other sequences collected globally and has no known associations with virulence or transmissibility. Sequences further clustered into 3 groups based on additional SNPs: cluster 1, a hospital cluster from the 2 largest hospitals in Danang (n = 9); cluster 2, a simultaneously detected community cluster within the center of Danang (n = 14); and cluster 3, a community cluster in the Son Tra district of Danang, detected August 6–8 (n = 3).

**Figure:**
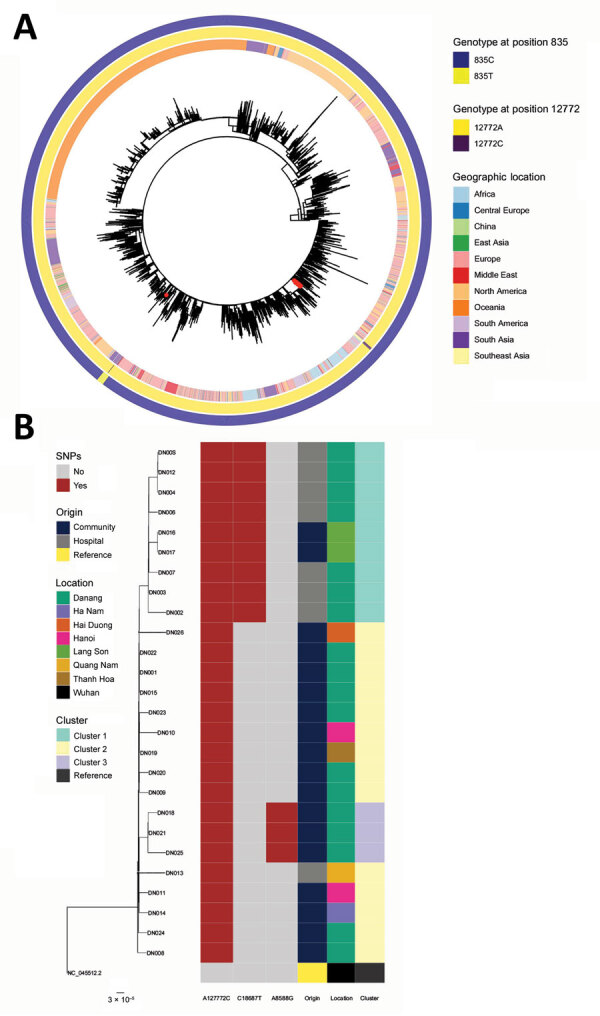
Maximum likelihood phylogenetic trees of SARS-CoV-2 B.1.1 lineage sequences globally and sequences from Danang, Vietnam. A) Global maximum-likelihood phylogenetic tree of SARS-CoV-2 B.1.1 lineage. The phylogeny was inferred with the general time-reversible plus frequencies model using 1,000 bootstrap replicates. Red dots represent viruses from the Danang cluster. The outer ring shows lineage as determined using Pangolin (https://github.com/cov-lineages/pangolin/releases/tag/v2.3.0), and the inner ring shows the geographic location of collection. B) Maximum-likelihood phylogenetic tree built from 26 Danang-related SARS-CoV-2 sequences (represented by DN plus a 3-digit number); the Wuhan strain genome (GenBank accession no. NC_045512.2) is an outgroup. Columns to the right show the nucleotide variation in 3 locations on the SARS-CoV-2 genome that define phylogenetic clusters in the Danang cluster with their origin, the location the patients were found, and the cluster of the sequence. The ModelFinder Plus option Hasegawa-Kishino-Yano substitution model, including modelling of amino acid frequencies was the best model for these samples. Scale bar indicates substitutions per site. SARS-CoV-2, severe acute respiratory syndrome coronavirus 2.

A synonymous mutation, C18687T, defines cluster 1 (Figure, panel B). Cases belonging to cluster 1 were mostly hospital-related (inpatients, caregivers, and workers). This mutation was later also found in 2 sequences (DN016 and DN017) from Lang Son Province in patients who had recently traveled to Danang but were not linked to the hospitals. Sample size and availability of clinical metadata are limited and therefore no conclusions about transmission route and associations with severity can be drawn.

Cluster 2 is a community cluster detected in the center of Danang and among epidemiologically linked cases across the country (Hai Duong, Ha Nam, Thanh Hoa, Quang Nam); sequences carry only the unique A12772C mutation associated with this outbreak. This cluster also contains case-patient DN013, with the additional C835T SNP. An epidemiologic link between the index patient, DN001 (cluster 1), and cases of cluster 2 has not been confirmed.

Cluster 3 is defined by an additional mutation, A8588G (K2775E in open reading frame 1ab), which was found in cases DN021, DN018, and DN025. Neither of these mutations was found among B1.1 sequences in the GISAID database.

The nonsynonymous A12772C mutation that all sequences shared, which acted as a biomarker to determine the relatedness of cases found within and outside of Danang, was not found in the GISAID database or elsewhere. This finding suggests that a single introduction of SARS-CoV-2 of unknown origin with potential undetected circulation in the community before detection in case DN001 was responsible for this cluster.

The lack of community cases found in Vietnam for 99 days before this cluster, the observed high level of nucleotide identity (99.96% minimum, 99.97% median), and the presence of a unique shared mutation indicate that this virus was unlikely to have been circulating undetected in the community since April 2020 or that this outbreak was caused by multiple importations. Given the very low circulation of SARS-CoV-2 and restricted entry, contact tracing and quarantining of contacts on the basis of exposure rather than symptoms remain effective measures to prevent and contain circulation of SARS-CoV-2 in Vietnam.
